# Land Use Change Disrupts the Network Complexity and Stability of Soil Microbial Carbon Cycling Genes Across an Agricultural Mosaic Landscape

**DOI:** 10.1007/s00248-024-02487-9

**Published:** 2025-01-07

**Authors:** Alexa K. Byers, Steve A. Wakelin, Leo Condron, Amanda Black

**Affiliations:** 1 Bioprotection Aotearoa, Lincoln University, P.O. Box 85084, Lincoln, 7647 New Zealand; 2 Scion, Riccarton, Christchurch, 8011 New Zealand; 3 Faculty of Agriculture and Life Sciences, Lincoln University, P.O. Box 85084, Lincoln, 7647 New Zealand

**Keywords:** Land use change, Agricultural disturbance, Microbial network analysis, Soil carbon, Soil microbiome, Soil metagenomics

## Abstract

To understand the effects of agricultural land use change and management on soil carbon (C) cycling, it is crucial to examine how these changes can influence microbial soil C cycling. Network analysis can offer insights into the structure, complexity, and stability of the soil microbiome in response to environmental disturbances, including land use change. Using SparCC-based co-occurrence networks, we studied how land use change impacts the connectivity, complexity, and stability of microbial C-cycling gene networks across an agricultural mosaic landscape in Canterbury, New Zealand. The most densely connected networks were found in land uses that were under the most intensive agricultural management, or under naturally regenerating vegetation. The microbial C-cycling gene networks from both land uses presented high network connectivity, low modularity, and a low proportion of negative gene interactions. In contrast, microbial C-cycling genes from native forests, which had the most stable and undisturbed plant cover, had the lowest network connectivity, highest modularity, and a greater proportion of negative gene interactions. Although the differences in total soil C content between land uses were small, the large effects of land use on the network structure of microbial C-cycling genes may have important implications for long-term microbial soil C cycling. Furthermore, this research highlights the value of using microbial network analysis to study the metabolic gene interactions shaping the functional structure of soil microbial communities in a manner not typically captured by more traditional forms of microbial diversity analysis.

**Supplementary Information:**

The online version contains supplementary material available at 10.1007/s00248-024-02487-9.

## Introduction

Agricultural intensification and land use change are driving major shifts in the biodiversity, functioning, and resilience of soil ecosystems globally [1–3]. The importance of soil health to human well-being cannot be underestimated; soils provide multiple ecosystem services related to food and fibre production, air and water quality regulation, erosion and flood control, nutrient cycling, and greenhouse gas regulation [4]. Indeed, many of the United Nations Sustainable Development Goals are directly reliant on plant production and other processes supported by soils [5]. One major soil function impacted by agricultural land use change is soil carbon (C) storage [6]. Globally, soil ecosystems store the largest pool of terrestrial organic C in the biosphere [7]. Protecting and increasing these C pools is essential for regulating the balance of the global C cycle and mitigating the effects of rising atmospheric CO_2_ emissions [8]. Moreover, soil C is important for the maintenance of soil structure, water and nutrient retention, and water and nutrient use and efficiency. Consequently, the quantity, quality, and cycling of the soil C pool are important indicators of soil quality and health [9]. By driving major changes in fundamental ecosystem properties that influence soil C cycling, such as plant biodiversity and biomass, land use change can drive significant declines in the size and stability of the soil C pool [6]. To develop sustainable land use practices that support human needs while minimising depletion of the soil C pool, understanding the impacts of land use change on soil C cycling is critically important.

Soil microorganisms are responsible for the decomposition of soil organic matter [10] and, as such, significantly influence the rate and fate of soil C turnover and storage. Therefore, changes in the activity and functioning of soil microbial communities following land use change can impact soil C cycling [11]. When studying the response of soil microorganisms to environmental disturbances, it is important to note that they function within communities that are dynamically shaped by both positive (e.g. mutualistic) and negative (e.g. competitive) interactions, along with shared ecological niches and dispersal processes [12–14]. However, understanding how these microbial interactions are shaped by the complex system of hydrological, mineralogical, and physicochemical properties that comprise the soil milieu is, at best, challenging. The application of network analysis, which is based on patterns of species or gene co-occurrences within microbial communities, is increasingly used to identify important links between microbial communities and environmental factors such as soil chemistry and climate [15, 16], as well as to explore the structure and complexity within the microbiome itself [17]. Several key network properties (e.g. connectivity, modularity, robustness, and negative cohesion: positive cohesion) can be used to assess changes in the stability and resilience of microbial community interactions in response to environmental change [18–20]. Most previous studies have constructed microbial co-occurrence networks via the taxonomic profiles of soil microbial communities, as captured via 16S rRNA/ITS amplicon sequencing [15, 18, 21–23]. Despite the value of network analysis in exploring the functional structure of microbial communities [24], few researchers have applied microbial network analysis to investigate the interactions shared between microbial metabolic (sensu functional) genes related to essential soil ecosystem processes, such as soil microbial C-cycling genes.

This research aimed to examine the effects of land use change on networks of soil microbial genes associated with C cycling pathways across a productive agricultural landscape. Five land uses covering a gradient of high-to-low land-use intensity were targeted for investigation, spanning sites of pastoral agriculture through to native forest. By analysing differences in the structure, complexity, and stability of microbial C-cycling gene networks across different land uses, we aimed to better understand the potential impacts of land use change on microbial soil C cycling. Research has shown that land use intensification (e.g. land-use conversion, synthetic fertilizer and pesticide application, irrigation, mechanical soil disturbance, removal of native plant biodiversity, and intensive monocropping) can increase the complexity and stability of soil microbial communities [20, 22, 25]. Based on these findings, we hypothesised that these effects would also be observed at the functional level. That is, we would observe an increase in the network complexity of microbial functional genes related to major C cycling metabolic potential in the soil metagenome.

## Methods

### Soil Sampling

Soils were collected from different land uses across an agricultural mosaic landscape in Canterbury, Aotearoa New Zealand (Kaituna Valley and Prices Valley). This was part of a study originally described by Byers et al. [26], and the methods, including soil chemical analysis, DNA extraction, and metagenomics sequencing, have been described fully in this previous study. Briefly, five land uses were selected for sampling that covered a gradient from low to high intensity and included (1) native forest, (2) regenerating native bush (‘regenerating bush’), exotic *Pinus radiata* forest (‘exotic forest’), dryland sheep and dairy pasture (‘dryland pasture’), and irrigated sheep and dairy pasture (‘irrigated pasture’). The native forest sites represented the least disturbed sites and were small, remnant fragments of lowland podocarp forest [27, 28] containing native tree species such as tītoki (*Alectryon excelsus*), mahoe (*Melicytus ramiflorus*), mataī (*Prumnopitys taxifolia*), and kahikatea (*Dacrycarpus dacrydioides*). The regenerating bush and exotic forest sites were areas historically cleared for pastoral grazing that either regenerated into patches of native bush dominated by kānuka (*Kunzea ericoides*) or were planted into exotic *Pinus radiata* forest (post-1996 to 2001). The regenerating bush and exotic forest sites represented land uses with less intensive agricultural management but with a high degree of historic disturbance. The irrigated and dryland pasture sites represented land uses under intensive agricultural management regimes, including sheep and dairy grazing and trampling, artificial fertiliser application, and irrigation.

Following surveys across the area, three sampling *sites* were identified each for native forest and exotic forest, and five sampling sites were identified each for irrigated pasture, dryland pasture, and regenerating bush. In each replicate site, five sampling *points* were marked out across a 5 m^2^ grid. At each sampling point, four soil cores were collected to a 10 cm depth (A horizon) following removal of the litter layer and combined to provide a 500 g composite sample per sampling point. Five soil samples were collected per replicate site for each land use; three were selected for inclusion in this study, and two were stored for aligned research. Thus, there were 9 total samples for native forest and exotic forest (3 sites × 3 sampling points per site) and 15 total samples for regenerating bush, dryland pasture, and irrigated pasture (5 sites × 3 sampling points per site). The moist soils were sieved to 2 mm, and the subsamples were stored at − 20 °C for DNA extraction or at 4 °C until chemical analysis.

### Soil Chemical Analysis

All samples were analysed for pH, Olsen phosphorus (Olsen P; mg l^−1^), total carbon (total C; %), total nitrogen (total N; %), the C/N ratio, the ratio of anaerobically mineralizable N to total N (AMN/TN ratio), moisture content (%), cation exchange capacity (me 100 g^−1^), and the exchangeable cations calcium, magnesium, sodium, and potassium (Ca^2+^, Mg^2+^, Na^+^, and K^+^). The results of the soil chemical analysis are presented in Table S1 (Supplementary File).

### DNA Extraction, Metagenomic DNA Sequencing, and Bioinformatics Processing

Soil DNA was extracted from 0.25 g of each soil sample via the DNeasy PowerSoil Pro Kit (Qiagen). Shotgun metagenomics was performed via the Illumina NovaSeq PE150 platform (NovogeneAIT Genomics, Singapore). Paired-end metagenomic reads were co-assembled via MEGAHIT v1.2.9 [29]. Metagenome binning was performed via metaBAT2 v2.12.1 and MaxBin2 v2.2.6 binning algorithms using metaWRAP v1.2.1 [30]. METABOLIC v4.0 was used to predict protein-coding genes and calculate gene coverage values, which were standardised by read depth and scaled by average library size via SAMtools v1.15.1 [31, 32]. Protein-coding genes were annotated against the carbohydrate-active enzymes database (CAZy) to identify genes involved in the breakdown, modification, or biosynthesis of carbon [33]. CAZy annotation allocated genes to the major enzyme classes of glycosyltransferases (GTs; biosynthesis), glycoside hydrolases (GHs; degradation), polysaccharide lyases (PLs; degradation), carbohydrate esterases (CEs; degradation), auxiliary activities (AAs; assisting degradation), and carbohydrate-binding modules (CBMs; recognition/binding).

### Network Analysis

Before network construction, the dataset of standardised gene abundances was split by land use and filtered to remove low-abundance genes (fewer than 3 counts in less than 10% of the samples). A correlation-based network was constructed for each land use via the *microeco* R package [34]. To account for the compositional nature of the metagenomic survey data, the Sparse Correlations for Compositional data (SparCC) algorithm was used to infer correlations between microbial C-cycling genes [35]. Correlation significance was assessed through 1000 permutations. Correlations were filtered based on a coefficient threshold of 0.6 and an FDR-adjusted *p*-value of < 0.05. Links between nodes (i.e. individual CAZy genes) were weighted based on the correlation coefficient value. Network topological properties were calculated using the *meconetcomp* R package [36], including the total number of nodes (network size), total number of links (network connectivity), average node degree (average connectivity), average shortest path length, network diameter, average clustering coefficient, network density, network centralisation, and modularity. For detailed definitions of each topological property, see Table 1. Modularity, the degree to which a network can be divided into distinct sub-modules, was determined via fast greedy modularity optimisation [37]. The *microeco::cal_powerlaw()* function was used to determine whether the degree distribution of each network followed a scale-free, power law distribution [34]. Venn diagrams were constructed to identify the distribution of common genes between land uses [36]. Nodes were categorised as peripherals, connectors, module hubs, or network hubs based on their within-module (*z*_*i*_) and among-module connectivity (*P*_*i*_) (Table 1) [38]. To assess differences in network stability across land uses, network robustness, node vulnerability, and community cohesion were calculated via the *meconetcomp* R package using the *robustness()*, *vulnerability()*, and *cohesion()* functions [15, 19, 36]. Robustness was calculated by measuring the changes in network efficiency (‘*Eff*’) as nodes were deleted at 10% intervals until only 50% of the original nodes remained [36, 39]. Nodes were either deleted at *random* or *targeted* based on node-degree, whereby high-degree nodes were progressively deleted in descending order. To calculate network robustness using *random* node deletion, 100 iterations of random attack were performed. For more detailed definitions of each stability measure, see Table 1. Module eigengene analysis was performed to analyse the higher-order organisation of each network, and soil chemical properties were correlated with the module eigengenes to assess the influence of environmental parameters on network structure [15, 17]. Network structures were visualised via the *igraph* and *ggraph* R packages [40, 41].

**Table 1:** The network topological properties that were used to compare differences in the structure, complexity, and stability of soil microbial C-cycling gene networks between different land uses. General guidance for each definition was provided by Csárdi et al. [40] and Deng et al. [17], alongside the specific citations provided in-text

Network property	Definition
Network size	The total number of nodes in the network. Each node represents a microbial C-cycling gene classified according to the carbohydrate-active enzyme (CAZy) database [33]. *Thus, all instances of the term ‘node(s)’ in this study in fact refer to microbial C-cycling gene(s)*
Network connectivity	The total number of links shared between all nodes in the network. Each link represented a SparCC correlation coefficient of greater than 0.6 with an FDR-adjusted *p*-value of less than 0.05
Average connectivity	The mean number of links per node, also referred to as the average node degree
Average shortest path length	The mean length of all the shortest paths between all pairs of nodes in the network. A smaller average shortest path length indicates greater network connectivity, as the length required to travel between any two connected nodes is smaller
Network diameter	The length of the longest average shortest path length in the network, i.e. the distance between the two most distant nodes in the network
Average clustering coefficient	Measures the probability that the adjacent nodes (sometimes referred to as ‘neighbours’) of a given node are connected. A higher clustering coefficient indicates a greater global node connectivity
Density	The ratio between the number of actual network links relative to the number of possible network links. A higher network density indicates a greater global node connectivity [15]
Centralisation	A measure of network-level centralisation calculated based on node-level centrality scores. A high network centrality value indicates that a small number of nodes hold a large proportion of the total network links
Modularity	A measure of network community structure and the degree to which the network is compartmentalised into different modules [15]. Modules are subsets of nodes that are more highly correlated or anti-correlated with each other than the rest of the nodes present in the community. Higher modularity values are associated with greater network robustness, as the effects of changes to the abundance of one node are more limited to that node’s module rather than the entire set of nodes present in the community [19]
Node topological role—peripheral, connector, module hub, or network hub	Each node was assigned a topological role based on its within (*z*_*i*_) and among-module connectivity (*P*_*i*_). Peripherals are nodes with very few links within their modules (low *z*_*i*_, low *P*_*i*_). Connectors are nodes that connect different modules within the network (low *z*_*i*_, high *P*_*i*_). Module hubs are nodes that are highly connected within their module (high *z*_*i*_, low *P*_*i*_). Network hubs are nodes that are highly connected across the entire network (high *z*_*i*_, high *P*_*i*_) [38]
Network robustness	The ability of the network to maintain system functioning following the *random* or *targeted* deletion of nodes. In the context of this study, this would reflect either random gene loss, or the loss of highly connected genes that shared many correlations with other genes in the network [36, 39]
Node vulnerability	A vulnerability score for each individual node in the network calculated by measuring the decrease in network efficiency following the deletion of a node and all its associated links from the network. This metric provides a measure of how important each node is for the spread of ‘information’ amongst nodes present in the community, as greater losses in network efficiency following the deletion of a single node will have a greater impact on global network connectivity [15]
Network cohesion	An abundance-weighted measure of node connectivity calculated for each sample in the network [42]. Three measures of network cohesion were calculated—positive, negative, and negative: positive cohesion ratio [19, 42]. Positive cohesion was calculated based on the mean weight of nodes sharing positive correlation coefficients, and negative cohesion was calculated based on the mean weight of nodes sharing negative correlation coefficients. Negative cohesion values were converted to absolute negative values. The negative: positive cohesion ratio is the proportion of negative to positive interactions. A higher negative: positive cohesion ratio indicates a greater influence of negative ‘interactions’ between nodes in the network

## Results

### Network Topology

**Fig. 1:**
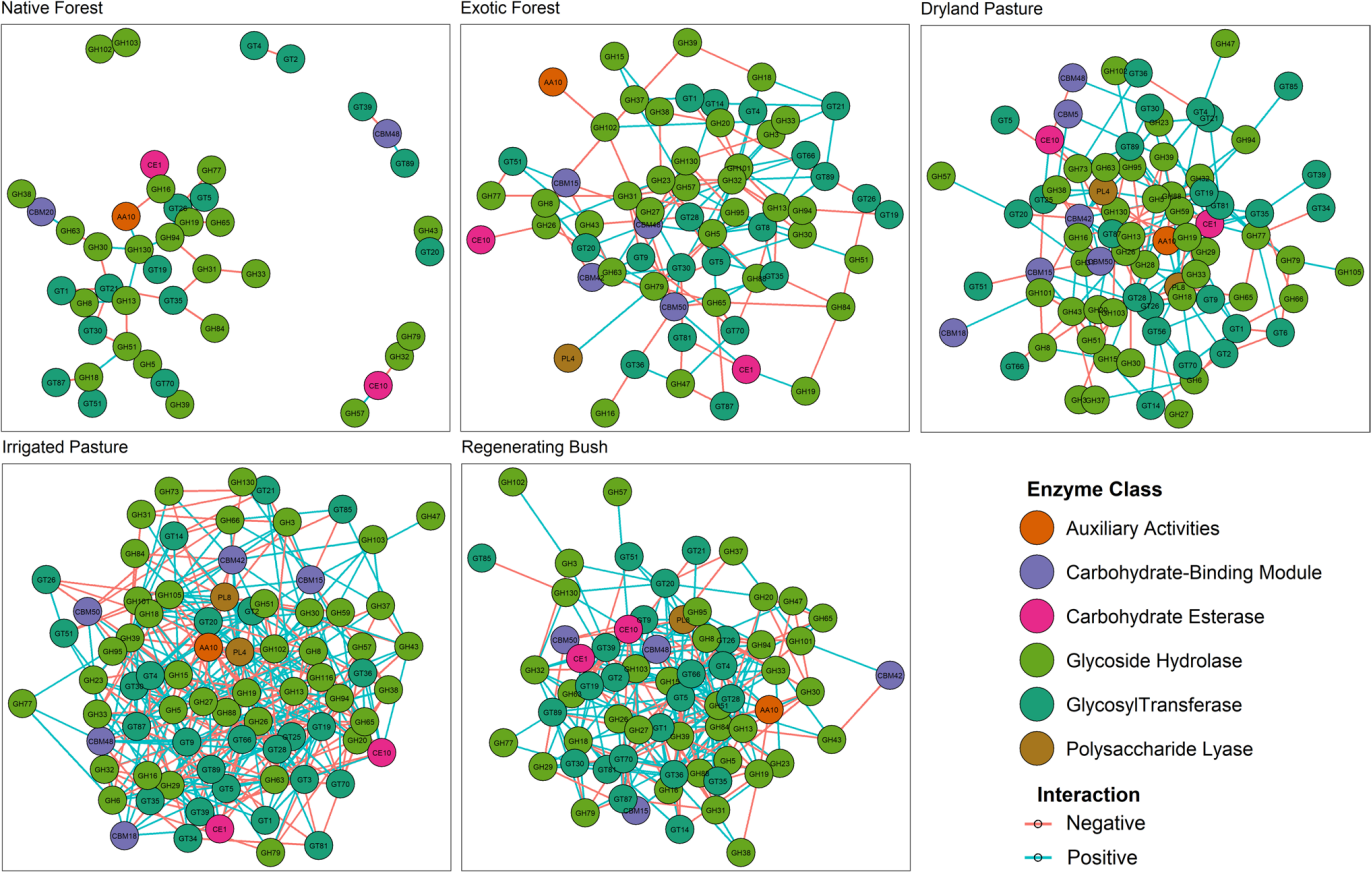
Co-occurrence networks of soil microbial C-cycling genes in land uses of exotic forest, native forest, dryland pasture, irrigated pasture, and regenerating bush. Nodes represent microbial C-cycling genes classified according to the carbohydrate-active enzyme (CAZy) database and are coloured based on their enzyme class. Edges between nodes represent their SparCC-based correlation coefficient (|*r*|> 0.6, FDR *p*-adjust. < 0.05). Positive correlations between nodes are coloured in blue, and negative correlations are coloured in pink

The microbial C-cycling gene networks in irrigated pasture and regenerating bush had the highest total and average connectivity, highest clustering coefficient, lowest average path length, and highest network density (Table 2). In contrast, the network of microbial C-cycling genes in native forest was sparser and less connected, with the lowest connectivity and density, largest network diameter, and longest average path length (Fig. 1, Table 2). For example, the average network connectivity of microbial C-cycling genes in irrigated pasture and regenerating bush was approximately four times greater than that in native forest. Overall, these differences in network topological structure indicate weaker metabolic gene interactions between microbial C-cycling genes in native forest compared to other land uses. The topological properties of networks in exotic forest and dryland pasture were intermediate relative to those in the other land uses, with the two land uses sharing similar values for average network connectivity, average path length, diameter, density, and centralisation (Table 2).

**Table 2:** The topological properties of soil microbial C-cycling gene networks associated with different land uses

Network property	Exotic forest	Native forest	Dryland pasture	Irrigated pasture	Regenerating bush
Network size	62	44	78	77	66
Network connectivity	126	44	170	380	250
Average connectivity	4.06	2.00	4.36	9.87	7.58
% Negative links	46.83	65.91	39.41	45.26	40.80
Average shortest path length	2.26	3.28	2.08	1.48	1.63
Network diameter	4	7	4	3	4
Average clustering coefficient	0.12	0	0.07	0.18	0.14
Network density	0.07	0.05	0.06	0.13	0.12
Network centralisation	0.15	0.07	0.13	0.17	0.13
Modularity	0.45	0.66	0.44	0.26	0.27
*R*^2^ power law	0.12	0.10	0.19	0.09	0.07
Peripherals	74%	82%	77%	43%	41%
Connectors	26%	18%	22%	57%	59%
Module hubs	0%	0%	1%	0%	0%
Network hubs	0%	0%	0%	0%	0%

The microbial C-cycling genes in irrigated pasture and regenerating bush had the lowest network modularity; the highest network modularity was observed in native forest (Table 2). Modularity measures the degree to which the network is compartmentalised into different subsets of highly correlated nodes [19]. No networks from any land use had a significant fit with the power-law distribution and did not follow a scale-free structure (Table 2). The microbial C-cycling genes in irrigated pasture had the highest network centralisation, driven by the influence of a few high-degree nodes that represented a high proportion of the network links. Both irrigated pasture and regenerating bush had a greater number of high-degree nodes, particularly compared to native forest (see networks presented in Figure S1 [Supplementary File], where network nodes are weighted by node degree).

When categorised according to their within (*z*_*i*_) and among (*P*_*i*_) network connectivity, most nodes in the exotic forest, native forest, and dryland pasture networks were peripherals, sharing few links with the rest of the network (Table 2; Fig. 1). Most nodes in irrigated pasture and regenerating bush networks were categorised as connectors, which are nodes responsible for connecting network modules. The greater proportion of connector nodes in irrigated pasture and regenerating bush aligns with the low modularity observed in these land uses, as there was a greater number of links connecting different modules (Table 2). In addition to module hubs, network hubs and connector nodes can be regarded as keystone nodes that are important for shaping the network structure [15].

### Node Composition

The networks of microbial C-cycling genes in irrigated and dryland pasture were the largest, having 75% more nodes than those in native forest (Table 2). When node overlap between land uses was compared, 47% of all nodes were found in every network (Figure S2, Supplementary File). The irrigated pasture and dryland pasture sites shared the greatest number of common nodes, with 9.6% of the nodes present only in these land uses. In addition, dryland pasture and irrigated pasture had the highest number of ‘unique’ nodes present in only one land use (4.9% and 2.4%, respectively). Despite sharing a similar network structure and topological attributes, exotic forest and regenerating bush shared no nodes exclusive to these land uses.

The most dominant enzyme classes of microbial C-cycling genes were glycoside hydrolases (≥ 53%) and glycosyltransferases (≥ 32%); these patterns were consistent across all land uses (Table S2, Supplementary File). Glycoside hydrolases and glycosyltransferases also occupied the highest proportion of links in each network, with the proportion of links occupied by at least one glycoside hydrolase ranging from 62.5% in native forest to 52.4% in regenerating bush. For glycosyltransferases, the proportion of links ranged from 27.3% in native forest to 37.2% in regenerating bush (Table S3, Supplementary File).

**Fig. 2:**
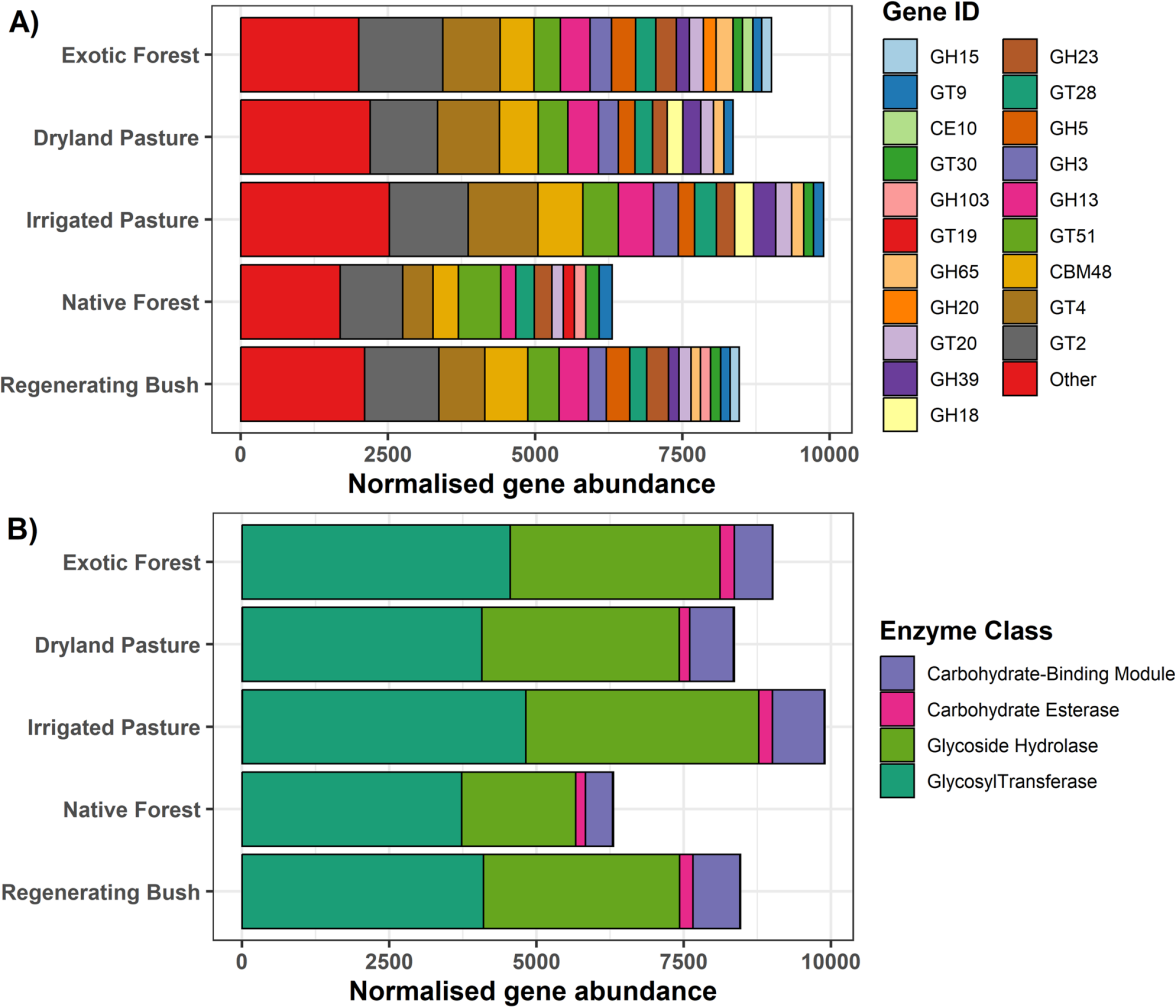
The normalised abundance of microbial C-cycling genes associated with each land use, represented by their **A** gene ID and **B** enzyme class (CAZy database). Owing to a high number of genes having a very low abundance, individual genes with a normalised abundance of less than 150 were grouped into ‘other’ for plot A

**Fig. 3:**
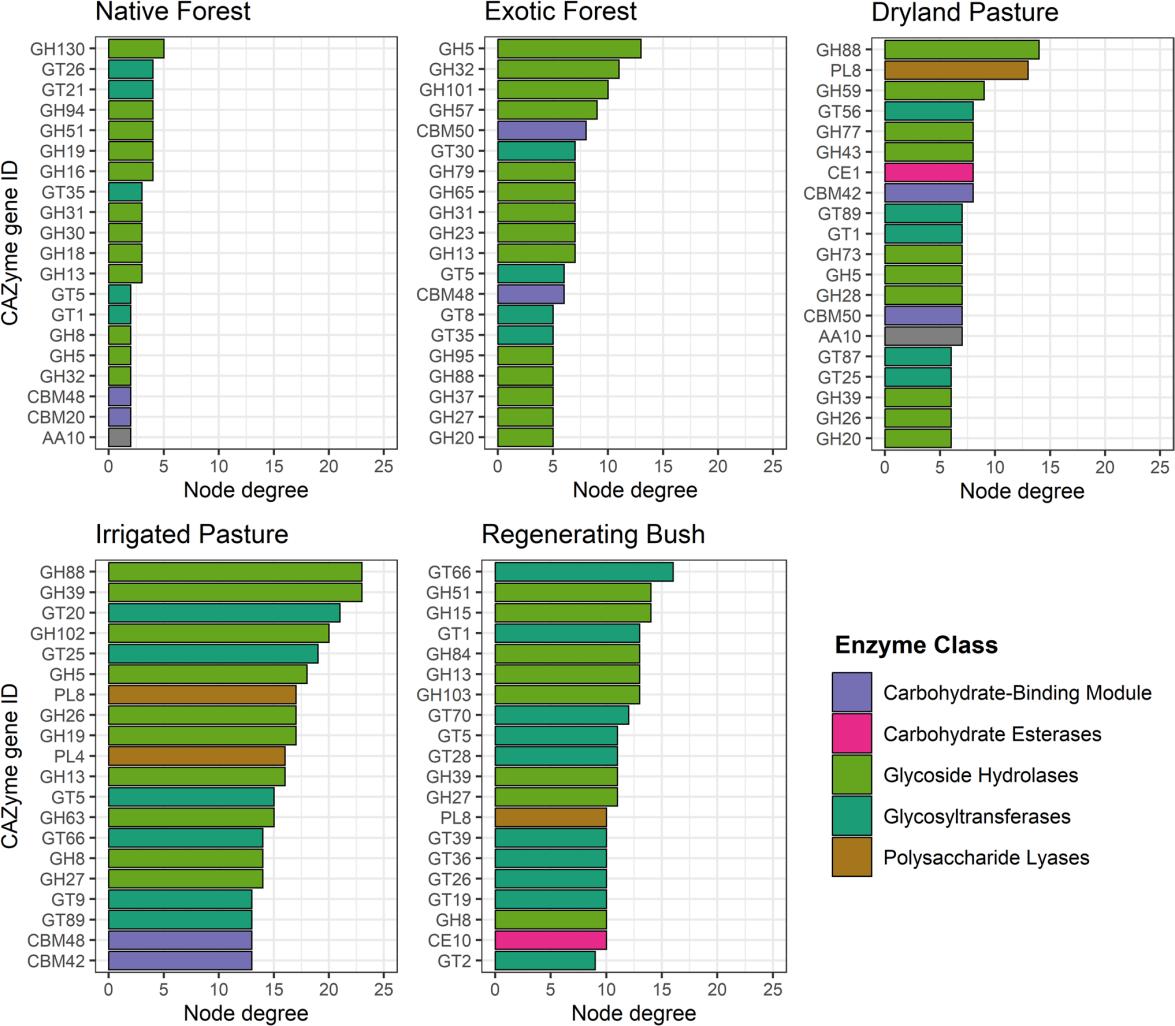
Among the microbial C-cycling genes with the highest node degree under each land use, only the top 20 genes are displayed. Microbial C-cycling genes are identified according to their gene ID and coloured according to their enzyme class (CAZy database)

Native forest had the lowest abundance of microbial C-cycling genes, and irrigated pasture had the highest, followed by exotic forest, regenerating bush, and dryland pasture (Fig. 2). The most abundant classes of microbial C-cycling genes were glycosyltransferases, glycoside hydrolases, and carbohydrate-binding modules (Fig. 2). Compared with the other land uses, native forest had a greater proportion of glycosyltransferases relative to glycoside hydrolases. Although the gene families GT2, GT4, CBM48, GT51, and GH13 were highly abundant across all the land uses (Fig. 2), they did not have a high node degree when fitted in the networks (Fig. 3). The nodes with the highest degree in irrigated pasture included the glycoside hydrolases GH88, GH39, GH102, and GH5 as well as the glycosyltransferases GT20 and GT25 (Fig. 3). In regenerating bush, nodes with the highest degree included the glycosyltransferases GT66, GT1, and GT70, as well as the glycoside hydrolases GH51, GH15, GH84, GH13, and GH103 (Fig. 3).

### Network Stability Analysis

**Fig. 4:**
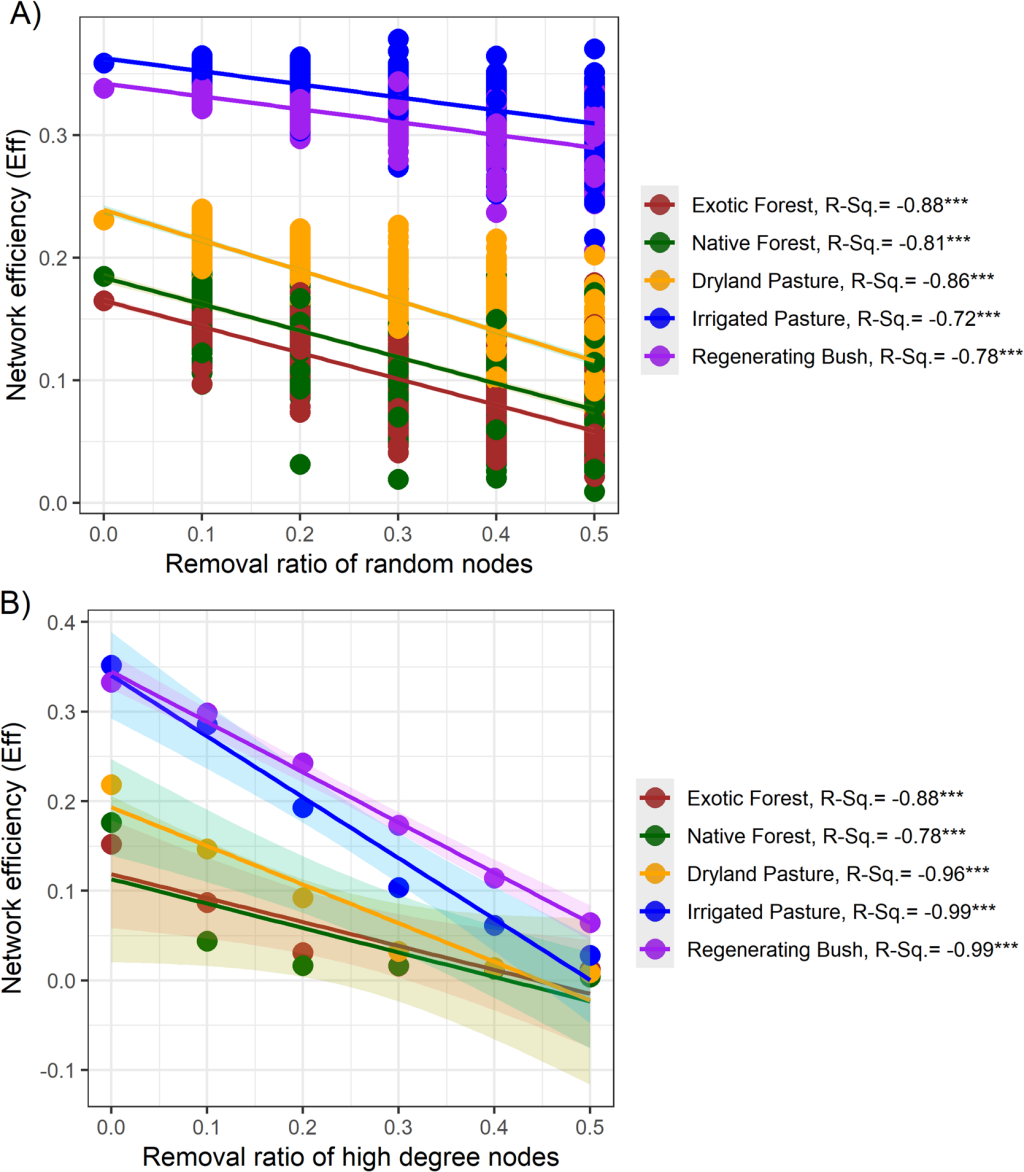
The changes in network robustness following either the **A** *random* or **B** *targeted* removal of 50% of network nodes, as measured via changes in network efficiency (Eff). *Targeted* node removal was performed based on node degree; whereby high-degree nodes were removed in descending order. Random node removal was performed using 100 iterations of random attack. The *R*^2^ values indicate the degree of decline in network robustness for each land use, and significant *p*-values < 0.001 are indicated by ***

All land uses displayed significant declines in network robustness following the *random* and *targeted* removal of 50% of the network nodes (Fig. 4). Exotic forest and dryland pasture presented the greatest declines in network robustness following *random* node removal (Fig. 4A). Irrigated pasture and regenerating bush displayed the smallest declines in network robustness under *random* node removal, but the largest declines occurred following *targeted* removal of high-degree nodes (i.e. nodes with the highest connectivity removed first; Fig. 4B). The large declines in network stability following *targeted* node removal may be due to these land uses having a greater number of high-degree nodes (Fig. 3, Figure S1, Supplementary File), as the loss of high-degree nodes and their associated links had a greater impact on global network connectivity. For example, the highest-degree node in native forests had only 5 links, whereas the highest-degree nodes in irrigated and regenerating bush had 23 and 16 links, respectively.

**Fig. 5:**
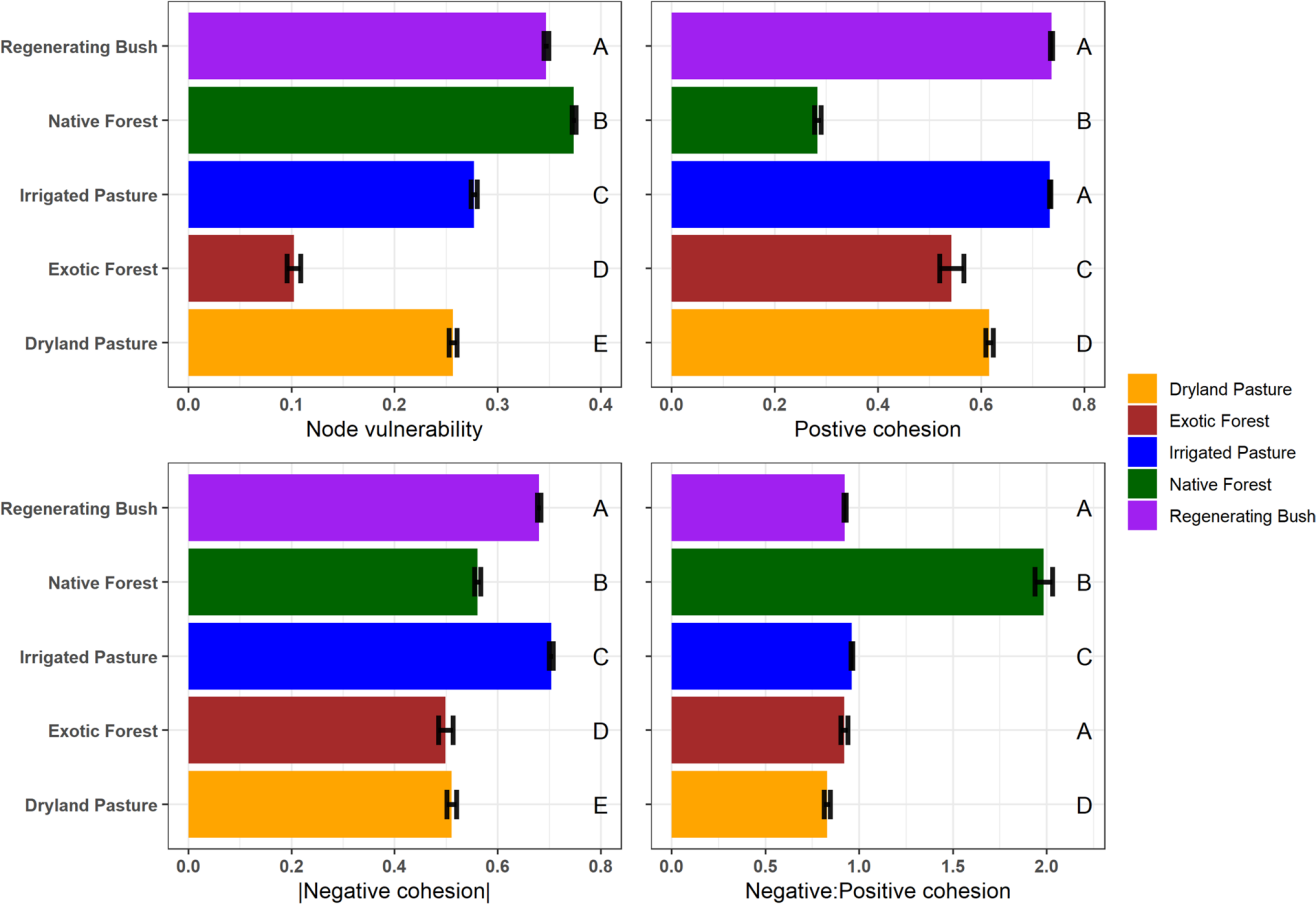
The average node vulnerability, positive network cohesion, absolute negative network cohesion, and negative: positive network cohesion ratio of the microbial C-cycling genes in each land use. The bars and error bars represent the mean ± SD values. Land uses not sharing any common letter were determined to be significantly different by the Tukey test at the 5% level of significance

There were significant differences in the average node vulnerability of microbial C-cycling genes between land uses (*F* = 46,485, *DF* = 4, *p* < 0.001; Fig. 5). Native forest had the lowest average node vulnerability, particularly compared with irrigated pasture and regenerating bush (Fig. 5). This may be related to the lower network connectivity and greater modularity observed in native forest, as the deletion of a single node had a lower impact on global network efficiency compared to land uses where nodes were more centralised and connected within the network. In contrast, networks with the highest node vulnerability (irrigated pasture and regenerating bush) had a greater number of high-degree nodes (Fig. 3, Figure S1, Supplementary File), as well as higher values for network centralisation and connectivity. Due to the greater number of links associated with the high-degree nodes observed in irrigated pasture and regenerating bush, the targeted removal of these high-degree nodes posed a greater risk to global network efficiency compared to land uses with a greater proportion of low-degree nodes, as the deletion of a single node had a greater impact on the overall spread of ‘information’ (mediated by node links) between genes in the microbial community.

Significant differences were observed between land uses across all measures of network cohesion, including positive cohesion (*F* = 3876, *DF* = 4, *p* < 0.001), negative cohesion (*F* = 2012, *DF* = 4, *p* < 0.001), and the negative: positive cohesion ratio (*F* = 5043, *DF* = 4, *p* < 0.001). Despite regenerating bush and irrigating pasture having the highest positive network cohesion, they also had the highest negative network cohesion (Fig. 5). This may be explained by the higher global network connectivity observed in these land uses, as the mean strength of the links was greater overall. Native forest had the highest relative strength of negative gene interactions, with the negative: positive cohesion ratio being significantly greater in native forest than in all other land use types (Fig. 5). In terms of link count, native forest also had the highest percentage of negative gene interactions, with more than 65% of the links between microbial C-cycling genes being negative correlations (Table 2). Under the assumption that links between nodes represent metabolic interactions between microbial C-cycling genes, these findings suggest that negative gene interactions had a greater influence on the network structure of microbial C-cycling genes in native forest than in other land uses.

### Influence of Soil Chemical Properties on the Network Structure of Microbial C-Cycling Genes

**Fig. 6:**
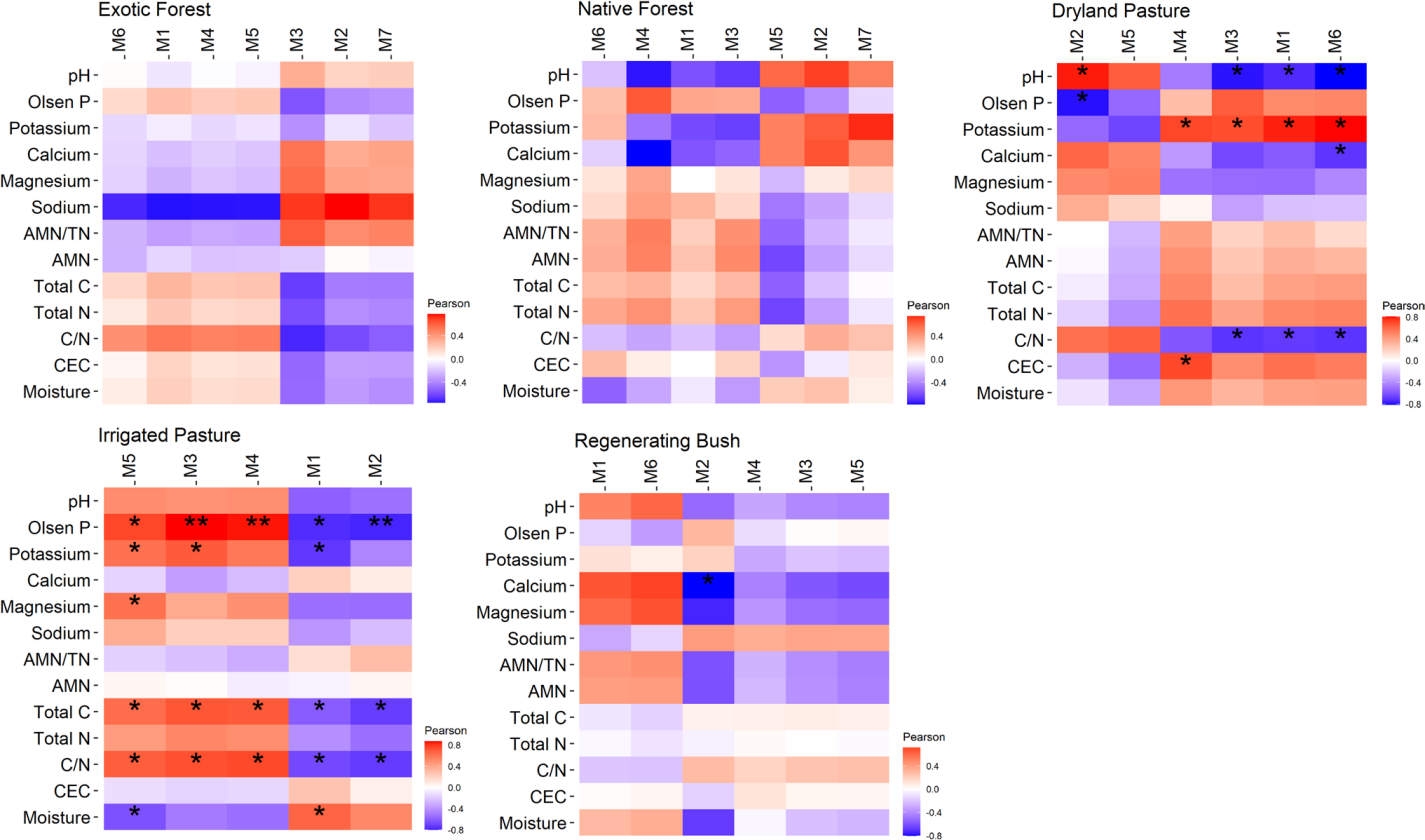
The Pearson correlations of network eigengene modules and soil chemical properties under each land use. Positive correlation coefficients are indicated in red, and negative correlation coefficients are indicated in blue. Significant FDR *p*-adjusted values are denoted by *, where * *p*-adj. < 0.05, ** *p*-adj. < 0.01, and *** *p*-adj. < 0.001

Module eigengene analysis revealed several soil chemical properties in dryland pasture and irrigated pasture that were significantly correlated with several of the network eigengene modules (i.e. were significant environmental factors associated with higher-order network organisation; Fig. 6). No soil chemical properties were significantly correlated with any network eigengene modules in native forest or exotic forest, and only one soil chemical property (calcium) was significantly correlated with one network eigengene module in regenerating bush (Fig. 6). These findings suggest that the influence of the soil chemical environment on the network structure of microbial C-cycling genes in native forest and exotic forest was lower than that in the other land uses.

## Discussion

This research revealed significant differences in the structure, connectivity, and stability of soil microbial C-cycling gene networks between different land uses across an agricultural mosaic landscape. Previous research has revealed inconsistent impacts of land use intensity and disturbance on microbial network complexity and connectivity. Although many studies have reported that land use intensity and disturbance decrease microbial network complexity and connectivity [14, 21, 23, 43], many other studies have reported the opposite trend [20, 22, 25]. As these previous studies focused on examining the taxonomic network structure of microbial communities, our research aimed to build on these studies by specifically examining the network structure of microbial functional genes related to soil C cycling. Thus, it is important to note any major discrepancies between our research findings and those of previous studies may also be due to differences in the gene region used to study microbial structure. In our study, irrigated pasture—the most intensively managed and productive land use—had the greatest network complexity, with the highest connectivity, clustering, density, centralisation, and network size. Despite having very different land use cover and management regimes, soil microbial C-cycling genes from regenerating bush also presented high network connectivity, complexity, and density. The land uses of regenerating bush investigated in this study were areas historically cleared for pastoral grazing but, owing to high rates of soil erosion and low productivity, were left to regenerate naturally with native plant species such as kānuka (*Kunzea ericoides*) [26]. The similarities in network structure between irrigated pasture and regenerating bush were unrelated to the influence of any common soil chemical properties. Despite the network organisation of irrigated pasture being strongly influenced by several soil chemical properties (e.g. Olsen P, total C, and the C/N ratio), the soil chemical environment had a weak influence on the higher-order network organisation of regenerating bush. Furthermore, the two land uses presented large differences in several key soil physiochemical properties. For example, irrigated pasture had a greater bioavailability of soil N and P (based on Olsen P and AMN values), a greater soil moisture content, and a far lower soil C/N ratio than soils associated with regenerating bush [26].

Considering the contrasting land use and management regimes associated with irrigated pasture and regenerating bush, it is difficult to identify what factors may be influencing the common attributes of their network structure (i.e. high complexity, connectivity, density, and centralisation). The aboveground environment associated with both land uses was subjected to more dynamic and frequent rates of change, whether through the effects of intensive agricultural management (e.g. artificial watering, fertiliser application, plant growth, and productivity) or naturally regenerating plant communities (e.g. greater flux in plant community composition and successional age). For example, previous research revealed that irrigation of dryland pasture sites in Canterbury (Aotearoa New Zealand) increased pasture production by up to 74% and stimulated increases in microbial respiration [44]. Whether through increased rates of plant productivity and/or frequent shifts in plant growth, community composition, and successional age, the increased rates of flux in the plant–root–soil environment of these land uses may have promoted more microbial interactions, including metabolic interactions related to soil microbial C-cycling. Land use duration has previously been reported to have a strong influence on microbial network structure [13], with network size, connectivity, modularity, and the proportion of negative gene interactions increasing with increasing land use duration. Replication of these findings was only partially observed in our study. We observed that microbial C-cycling genes in native forest, which had the most stable and oldest continuous plant community cover, presented the highest network modularity and proportion of negative gene interactions. However, native forest land use had the lowest network size and connectivity, which contradicts the effects of land use duration observed by Xu et al. [13]. To a lesser extent, these findings were also observed in exotic forest, which, despite having a longer land-use duration and greater plant maturity than the other land uses, had a relatively low network complexity and connectivity. Instead, we observed the largest and most complex microbial C-cycling gene networks in low-duration land use covers of irrigated pasture and regenerating bush, which was also associated with high temporal variation in aboveground management regimes and primary plant cover.

The different land uses displayed varying sensitivities to both random gene loss and the loss of highly connected metabolic pathways. For example, irrigated pasture and regenerating bush presented the greatest declines in network robustness after the targeted removal of high-degree nodes (i.e. highly connected genes), potentially indicating lower resilience to environmental disturbances [15, 18]. However, exotic forest and dryland pasture presented the greatest declines in network robustness following random removal of microbial C-cycling genes. Based on network robustness analysis alone, these discrepancies make it difficult to pinpoint specific land uses with the lowest resilience to environmental disturbances. However, microbial C-cycling genes from irrigated pasture and regenerating bush also presented many features typically associated with reduced network stability, such as high connectivity and centrality, a low modularity and average path length, and a high proportion of positive gene interactions [45]. In an ecological context, these network properties indicate that the impacts of environmental disturbances spread more quickly through the metabolic interactions shaping the microbial community, potentially having a greater impact on microbial C-cycling [19].

In contrast, microbial C-cycling genes in native forest presented many features of a highly stable network, with the highest network modularity, highest proportion of negative gene interactions, and lowest node vulnerability [19]. The lower network connectivity and higher modularity observed in native forest means that, in theory, the effects of environmental disturbances on the activity or functioning of microbial C-cycling genes are more restricted and spread more slowly through the network, thus having a more limited impact on the overall metabolic functioning of the microbial community [18, 19]. Native forest had the highest proportion of negative gene interactions—a network measure proposed by several previous studies as an important indicator of network stability [13, 19, 46, 47]. Networks dominated by positive interactions are considered more unstable because there is a greater risk that community members share similar environmental and nutritional requirements and display similar ecological responses to environmental change [14, 15, 19, 46]. In contrast, networks with a greater proportion of negative interactions may be more resilient to disturbance, as the effects of perturbation are less likely to rapidly spread through the community between microbes whose abundances are not positively associated [15, 19]. The greater proportion of negative gene interactions between microbial C-cycling genes in native forest may be indicative of environmental conditions such as nutrient limitation or low soil C bioavailability driving increased rates of microbial competition for soil C resources [15, 16]. This may be related to the greater abundance of woody plant biomass in native forest, potentially reducing the proportion of more readily available sources of soil C relative to more recalcitrant forms. In contrast, the greater proportion of positive gene interactions in intensively managed agricultural land uses (irrigated pasture) and historically disturbed land uses with actively regenerating plant communities (regenerating bush) may have a greater overlap of functional metabolic activity because the soil environment is dominated by a greater proportion of fast cycling, bioavailable soil C [19, 48, 49]. Previous research has proposed that ecological disturbance may increase microbial cooperation and metabolic cross-feeding [50], which may explain a greater proportion of positive gene interactions between soil microbial C-cycling genes in networks associated with higher-intensity land use.

Despite notable differences in the structure and stability of soil microbial C-cycling gene networks between land uses, these differences were not reflected in the total soil C content. Although the soil C content was lowest in agricultural land uses of irrigated pasture and dryland pasture, the differences between land uses were small and mostly non-significant, as reported by Byers et al. [26] (see also Table S1, Supplementary File). Furthermore, across nearly all land uses, our findings revealed that the soil C content had a weak influence on the network structure of microbial C-cycling genes. Because we observed only minor differences in the soil C content between the different land uses, we are limited in concluding the implications of this study’s findings on long-term soil C storage. However, this study highlights the value of using microbial network analysis to better understand the metabolic gene interactions shaping soil microbial communities beyond traditional diversity metrics. Previous work carried out at the same sites revealed that the alpha diversity of microbial C-cycling genes in native forests was markedly lower than that in land uses associated with greater agricultural intensity [26]. The low diversity of microbial C-cycling genes in the native forest mirrored the patterns observed in the present study, where we observed that the native forest had a very small network size and low connectivity. However, the patterns that were mirrored between microbial alpha diversity and network complexity in native forest were not detected across all land uses. The alpha diversity analysis performed by Byers et al. [26] did not reveal the distinctive profile of microbial C-cycling genes associated with sites of regenerating bush land uses. Compared with those in native forests, the alpha diversity of microbial C-cycling genes in regenerating bush was lower than that in agricultural land uses. However, microbial C-cycling genes in regenerating bush have complex and densely connected network structures, sharing many network properties like those of irrigated pasture. These findings highlight the value of performing microbial network analysis to provide deeper insights into the complex metabolic gene interactions shaping microbial community structure that cannot typically be captured based on microbial diversity analysis alone.

## Supplementary Information

Below is the link to the electronic supplementary material.

Supplementary file1 (DOCX 452 KB)

## Data Availability

The datasets generated during and/or analysed during the current study are available from the corresponding author upon reasonable request.
