# Supplementary material for: Land Use Change Disrupts the Network Complexity and Stability of Soil Microbial Carbon Cycling Genes Across an Agricultural Mosaic Landscape
Source: Microb Ecol. 2025 Jan 7;87(1):167. doi: 10.1007/s00248-024-02487-9 (PMC11706911; doi:10.1007/s00248-024-02487-9)
Supplement: Supplementary file 1 — Supplementary file1 (DOCX 452 KB) [file 248_2024_2487_MOESM1_ESM.docx]

**Supplementary File**

**Land use change disrupts the network complexity and stability of soil microbial carbon cycling genes across an agricultural mosaic landscape**

Alexa K. Byers^1^, Leo Condron^2^, Steve A. Wakelin^3^, Amanda Black^1^

^1^Bioprotection Aotearoa, P.O. Box 85084, Lincoln University, Lincoln 7647, New Zealand

^2^Faculty of Agriculture and Life Sciences, P.O. Box 85084, Lincoln University, Lincoln 7647, New Zealand

^3^Scion, PO Box 29237, Riccarton, Christchurch, 8440, New Zealand

Corresponding author: Alexa K. Byers ([alexa.byers@lincoln.ac.nz](mailto:alexa.byers@lincoln.ac.nz))

**Table S1. The mean ± SD values for the soil chemical properties in each land use. The results of Type-II Wald Chi-squared tests of linear mixed-effects models are presented, which were used to test the fixed effect of land use on soil chemical properties. Land uses not sharing a common letter were determined to be significantly different using multiple comparison tests.**

| **Soil property** | **Exotic Forest** | **Native Forest** | **Dryland Pasture** | **Irrigated Pasture** | **Regenerating Bush** | **Type II Wald Chi-square test results** |
| --- | --- | --- | --- | --- | --- | --- |
| pH | 5.1 ± 0.19 **b** | 6.12 ± 0.27 **a** | 5.92 ± 0.59 **a** | 5.91 ± 0.32 **a** | 5.57 ± 0.35 **ab** | Chi sq.= 15.72, p=0.03 |
| Olsen P (mg L^-1^) | 12.89 ± 10.62 **a** | 20.44 ± 8.89 **a** | 69.93 ± 57.41 **a** | 57.13 ± 24.99 **a** | 21 ± 18.66 **a** | Chi sq.= 11.24, p=0.024 |
| Total C (%) | 4.43 ± 1.72 **ab** | 4.97 ± 1.32 **ab** | 3.39 ± 0.74 **a** | 4.1 ± 0.67 **ab** | 5.31 ± 0.94 **b** | Chi sq.= 12.69, p=0.013 |
| Total N (%) | 0.31 ± 0.1 | 0.38 ± 0.09 | 0.34 ± 0.08 | 0.42 ± 0.06 | 0.38 ± 0.07 | Chi sq.= 7.75, p=0.101 |
| C/N ratio | 14.08 ± 1.25 **a** | 12.92 ± 1.07 **a** | 10 ± 0.53 **b** | 9.77 ± 0.51 **b** | 14.01 ± 0.79 **a** | Chi sq.= 178.0, p<0.001 |
| AMN (µg/g) | 42.56 ± 8.34 **c** | 200.9 ± 107.6 **a** | 121.5 ± 61.3 **bc** | 193.4 ± 47.8 **a** | 85.33 ± 33.2 **ab** | Chi sq.= 36.26, p<0.001 |
| AMN/TN ratio | 1.47 ± 0.35 **b** | 4.98 ± 1.48 **a** | 3.39 ± 1.09 **b** | 4.61 ± 1.05 **a** | 2.26 ± 0.85 **b** | Chi sq.= 62.23, p<0.001 |
| CEC (me 100g^-1^) | 18.67 ± 3.84 **bc** | 25.78 ± 3.9 **a** | 17.93 ± 2.7 **b** | 22.27 ± 1.94 **ac** | 21 ± 2.9 **bc** | Chi sq.= 27.84, p<0.001 |
| Potassium (% BS) | 1.57 ± 0.31 **a** | 2.02 ± 0.34 **a** | 7.04 ± 3.35 **b** | 4.47 ± 1.98 **ab** | 2.88 ± 1.08 **a** | Chi sq.= 27.60, p<0.001 |
| Calcium (% BS) | 17.78 ± 3.31 **b** | 44.56 ± 4.9 **a** | 51.57 ± 12.02 **a** | 45.2 ± 6.3 **a** | 24.6 ± 8.8 **b** | Chi sq.= 51.56, p<0.001 |
| Magnesium (% BS) | 14.29 ± 4.73 **b** | 25.2 ± 1.33 **a** | 13.81 ± 5.42 **b** | 20.93 ± 2.04 **a** | 19.51 ± 6.92 **ab** | Chi sq.= 20.53, p<0.001 |
| Sodium (% BS) | 1.84 ± 0.34 **a** | 1.93 ± 0.73 **a** | 1.5 ± 0.33 **a** | 3.49 ± 0.97 **b** | 3.23 ± 0.81 **b** | Chi sq.= 47.97, p<0.001 |
| Moisture (%) | 6.18 ± 1.3 **b** | 24.03 ± 4.27 **a** | 14.4 ± 5.52 **c** | 18.51 ± 3.11 **ac** | 7.69 ± 2.61 **b** | Chi sq.= 47.64, p<0.001 |

*Note: The soil chemical properties presented in Table 1 were originally measured and reported by Byers, et al. [1].*

**
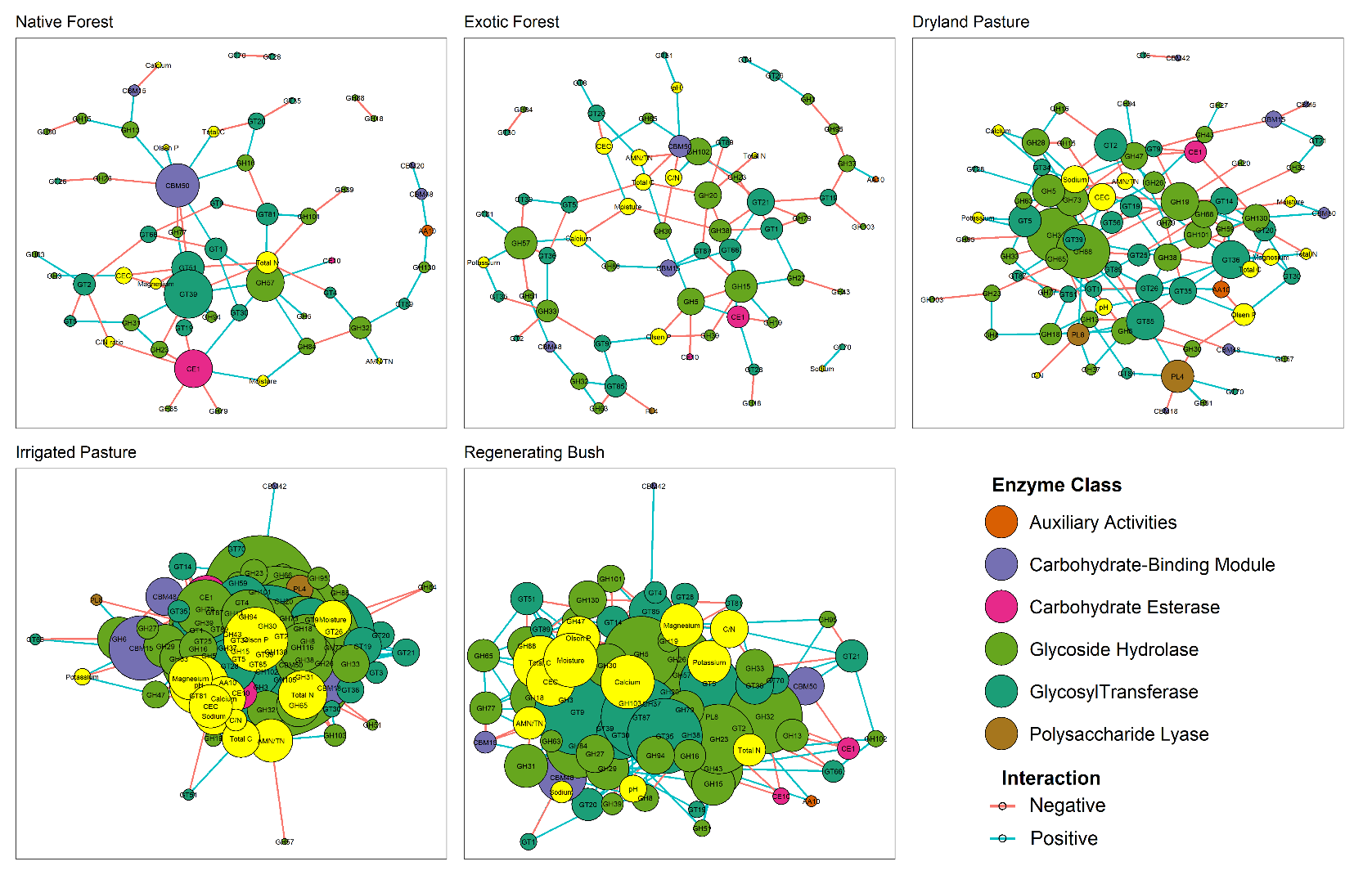
**

**Figure S1.** **Co-occurrence networks of soil microbial C cycling genes in land uses of exotic forest, native forest, dryland pasture, irrigated pasture, and regenerating bush. Nodes represent microbial C cycling genes classified according to the Carbohydrate-Active Enzyme (CAZy) database and are coloured based on their enzyme class. Edges between nodes represent their SparCC-based correlation coefficient (|r| > 0.6, FDR p-adjust. < 0.05). Node size is weighted by node degree i.e., total number of node edges (links). Positive correlations between nodes are coloured in blue and negative correlations are coloured in pink.**

**
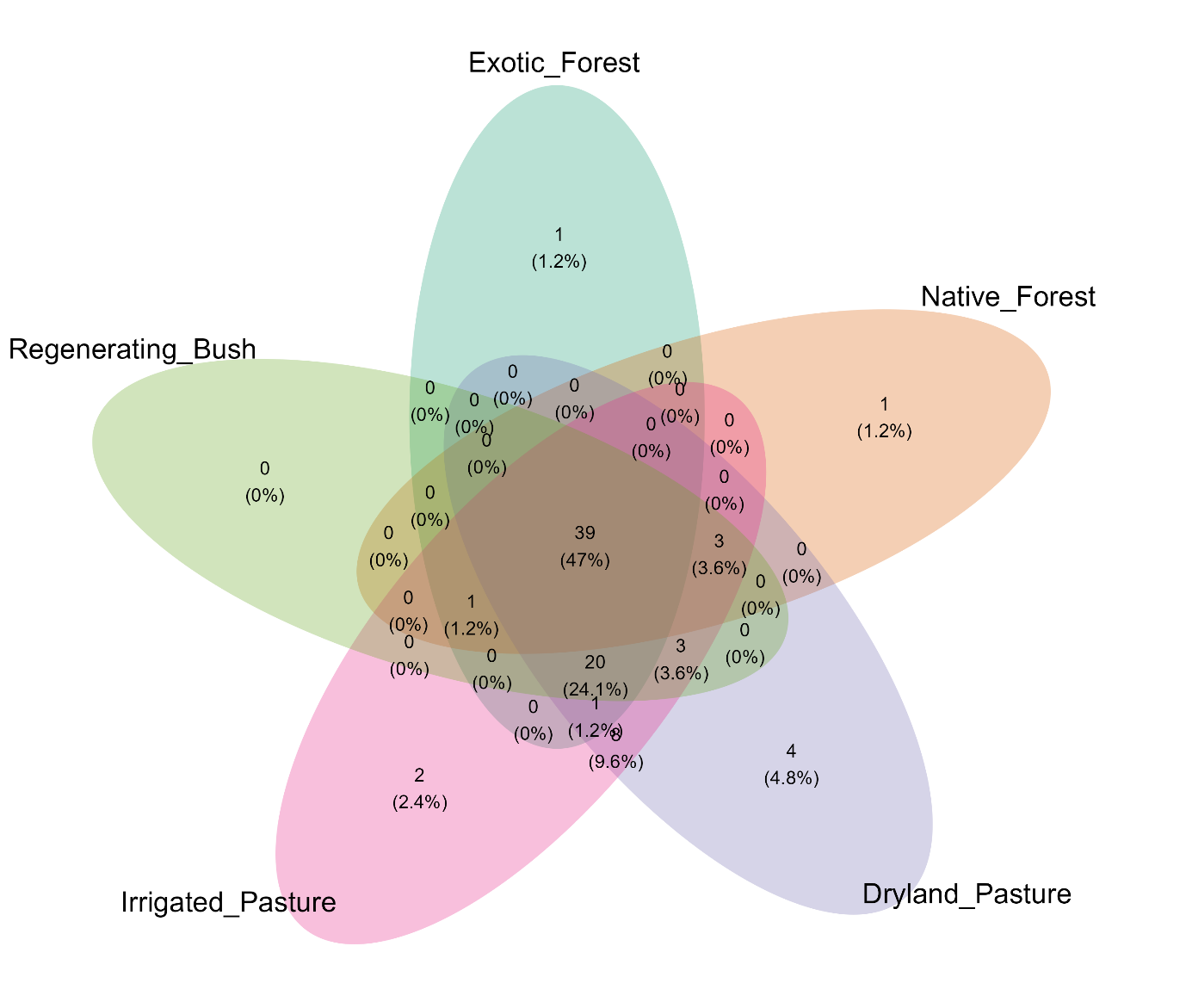
**

**Figure S2. The number and percentage of shared network between the five different land uses.**

**Table S2. The count and proportion (%) of network nodes representing different CAZyme gene enzyme classes in each land use.**

| **Gene type** | **Exotic Forest** | **Native Forest** | **Dryland Pasture** | **Irrigated Pasture** | **Regenerating Bush** |
| --- | --- | --- | --- | --- | --- |
| Auxiliary Activity | 1 (2%) | 1 (2%) | 1 (1%) | 1 (1%) | 1 (2%) |
| Carbohydrate Binding Module | 4 (6%) | 2 (5%) | 6 (8%) | 5 (6%) | 4 (6%) |
| Carbohydrate Esterase | 2 (3%) | 2 (5%) | 2 (3%) | 2 (3%) | 2 (3%) |
| Glycoside Hydrolase | 34 (55%) | 24 (55%) | 41 (53%) | 42 (55%) | 36 (55%) |
| Glycosyltransferase | 20 (32%) | 15 (34%) | 26 (33%) | 25 (32%) | 22 (33%) |
| Polysaccharide Lyases | 1 (2%) | 0 (0%) | 2 (3%) | 2 (3%) | 1 (2%) |

**Table S3. The % share of network links of different CAZyme gene enzyme classes in each land use.**

| **Enzyme Class** | **Exotic Forest** | **Native Forest** | **Dryland Pasture** | **Irrigated Pasture** | **Regenerating Bush** |
| --- | --- | --- | --- | --- | --- |
| Auxiliary Activity | 0.40 | 2.27 | 2.06 | 1.45 | 1.20 |
| Carbohydrate Binding Module | 8.33 | 4.55 | 7.35 | 6.58 | 4.00 |
| Carbohydrate Esterase | 1.59 | 3.41 | 3.53 | 2.37 | 3.20 |
| Glycoside Hydrolase | 61.90 | 62.50 | 52.65 | 53.42 | 52.40 |
| Glycosyltransferase | 27.38 | 27.27 | 29.12 | 31.84 | 37.20 |
| Polysaccharide Lyases | 0.40 | 0.00 | 5.29 | 4.34 | 2.00 |

**References**

1. Byers AK, Condron L, Wakelin SA, Black A (2024) Land use intensity is a major driver of soil microbial and carbon cycling across an agricultural landscape. Soil Biology and Biochemistry 196: 109508. doi: <https://doi.org/10.1016/j.soilbio.2024.109508>
